# Hyper-Aerotolerant *Campylobacter coli* From Swine May Pose a Potential Threat to Public Health Based on Its Quinolone Resistance, Virulence Potential, and Genetic Relatedness

**DOI:** 10.3389/fmicb.2021.703993

**Published:** 2021-07-16

**Authors:** Jae-Ho Guk, Hyokeun Song, Saehah Yi, Jae-Uk An, Soomin Lee, Woo-Hyun Kim, Seongbeom Cho

**Affiliations:** College of Veterinary Medicine and Research Institute for Veterinary Science, Seoul National University, Seoul, South Korea

**Keywords:** aerotolerance, *Campylobacter coli*, genetic relatedness, quinolone resistance, swine, virulence potential, production stages

## Abstract

*Campylobacter*, a major foodborne pathogen, is susceptible to oxygen. Recently, aerotolerant *Campylobacter* with enhanced tolerance to aerobic stress has become a major concern in food safety. However, the aerotolerance of *Campylobacter coli* from pigs has not been studied extensively. Here, we sought to investigate the prevalence of *C. coli* across multiple swine groups in farms, including weaning, growing, and fattening pigs in production stages and pregnant sows. Additionally, we analyzed *C. coli* aerotolerance, quinolone resistance, virulence potential, and multilocus sequence typing (MLST) genotypes. Finally, we compared the characteristics of *C. coli* according to the aerotolerance levels. In total, we obtained 124 (66.3%) *C. coli* isolates from 187 swine fecal samples across six swine farms. The pathogen was prevalent in weaning (45.5%), growing (68.3%), and fattening (75.4%) pigs, and pregnant sows (66.7%). Hyper-aerotolerant HAT *C. coli* (13.7% of 124 isolates) was present in all swine groups, with the highest proportion in the pregnant sows (27.3%). All HAT isolates possessed diverse virulence-related genes such as *flaA*, *cadF*, *pldA*, *ceuE*, and *cdtA*. All *C. coli* isolates were resistant to quinolones, and 12 (10%) presented high-level ciprofloxacin resistance (MIC ≥ 32 μg/mL). The proportion of *C. coli* isolates with a high-level ciprofloxacin resistance was the highest in HAT *C. coli* (18.8%). Furthermore, six MLST sequence types (STs) (ST827, ST830, ST854, ST1016, ST1068, and ST1096) of swine-derived *C. coli* were in common with human-derived *C. coli* (PubMLST). The proportion of *C. coli* belonging to such shared STs at each aerotolerance level was the highest in HAT *C. coli* (HAT vs. oxygen-sensitive; OR = 3.13). In conclusion, quinolone resistance of *C. coli* may be distributed throughout in all swine groups in farms. HAT *C. coli* is likely to remain in pig farms and re-infect other pigs in the farms. Furthermore, swine-derived HAT *C. coli* could be transmitted to humans easily through the food chain owing to its aerotolerance, and it could pose a threat to public health owing to its high-level ciprofloxacin resistance and virulence. This study highlights the need to develop management practices that prevent the transmission of swine-derived HAT *C. coli* to humans.

## Introduction

*Campylobacter*, a major foodborne pathogen, causes gastrointestinal infections in humans, with symptoms such as diarrhea, fever, abdominal pain, vomiting, and prostration (Kaakoush et al., 2015; O’Kane and Connerton, 2017; Riedel et al., 2020). This bacterium harbors diverse virulence-related genes responsible for its motility, cell adhesion, colonization, invasion, iron uptake, cytotoxin production, secretion, and Guillain–Barré syndrome expression (Bacon et al., 2000; Ziprin et al., 2001; Datta et al., 2003; Bolton, 2015; Corcionivoschi et al., 2015; Koolman et al., 2015). While campylobacteriosis with gastrointestinal infections is usually self-limiting, it may also lead to severe illnesses including bacteremia, meningitis, irritable bowel syndrome, Guillain–Barré syndrome, or arthritis (Azrad et al., 2018; Centers for Disease Control and Prevention, 2019). Antibiotics are prescribed for clinical treatment of severe *Campylobacter* infections in humans (Tang et al., 2017; Centers for Disease Control and Prevention, 2019), and fluoroquinolones are the most frequently used (Sato et al., 2004; Sifré et al., 2015; Tang et al., 2020; Hull et al., 2021). However, an increasing trend of *Campylobacter* strains with fluoroquinolone resistance has become a major concern (Engberg et al., 2001; Moore et al., 2006; Sahin et al., 2015; Bolinger and Kathariou, 2017; Sproston et al., 2018).

*Campylobacter* is an obligate microaerophile and capnophile, optimally growing at low oxygen (5–10%) and high carbon dioxide (5–10%) concentrations (Bronowski et al., 2014). This bacterium is generally highly susceptible to atmospheric oxygen (Kaakoush et al., 2007). However, recently, the high prevalence of aerotolerant (AT) and hyper-aerotolerant (HAT) *Campylobacter jejuni* strains with enhanced tolerance to aerobic stress has been identified in retail chickens. Moreover, these strains cluster mostly into a few major multilocus sequence typing (MLST) clonal complexes that are often involved in human *Campylobacter* infections (Oh et al., 2015). In addition, most human clinical *C. jejuni* strains were reported to be HAT, with tolerance to multiple stress, including disinfectant, freeze-thaw, and heat treatments (Oh et al., 2018). In a previous study, we found HAT *Campylobacter coli* to be prevalent in duck carcasses and meat (Guk et al., 2019). Based on MLST sequence types (STs), most of the HAT *C. coli* strains from ducks are genetically related to *C. coli* isolates from humans (Guk et al., 2019). These findings suggest that the aerotolerance of *Campylobacter* is likely related with infections in humans. For these reasons, the aerotolerance of *Campylobacter* has become a major concern.

Pigs only become sub-clinically infected with *Campylobacter* spp., especially *C. coli*, making them a potential source of *Campylobacter* infections in humans through the consumption of contaminated pork meat (Mataragas et al., 2008; Kempf et al., 2017; Di Donato et al., 2020; Riedel et al., 2020). In addition, contamination of carcasses or meat with swine feces during processing or slaughtering can pose risks to food safety (Abley et al., 2012; Nisar et al., 2018; Di Donato et al., 2020). Considering these findings and the possibility that aerotolerance may be involved in human *Campylobacter* infections, studies on the aerotolerance of swine-derived *C. coli* are needed.

The aims of this study were: (1) to investigate the prevalence of *C. coli* in pigs in different swine groups, including pigs in the production stages (weaning, growing, and fattening stage) and pregnant sows, at pig farms; (2) to analyze the characteristics, such as aerotolerance, quinolone resistance, virulence, and genetic relatedness, of *C. coli* in different swine groups; and (3) to compare its quinolone resistance, virulence, and genetic relatedness according to the aerotolerance levels. This study is the first to elucidate the different characteristics of *C. coli* across swine groups in pig farms and according to the aerotolerance levels.

## Materials and Methods

### Isolation and Identification of *C. coli* From Swine

A total of 187 fresh swine fecal samples were collected from six swine farms from March 2018 to January 2019. The number of samples at each farm ranged from 26 to 34, and the number of samples from each swine group was as follows: 5–6 in the weaning pigs (4–12 weeks old), 8–11 in the growing pigs (12–20 weeks old), 8–11 in the fattening pigs (21–30 weeks old), and 5–6 in the pregnant sows. The number of swine fecal samples according to the swine groups in each farm is shown in Supplementary Table 1. Swine fecal samples were collected from healthy, randomly selected pigs, and the number of fecal samples collected for each swine group was determined considering the number of pigs per swine group in the farms. Each fecal sample was collected using a sterilized cotton swab, stored in a sterilized tube, and then transported from the swine farms to our laboratory on the same day. In addition, enrichment of the samples was performed to isolate *C. coli* immediately after the samples were transported. Except for the pregnant sows, which resided on the farms for breeding, most pigs were raised in these swine farms until the fattening stage and then shipped to slaughterhouses. The samples were classified into four different swine groups, including weaning pigs (*n* = 33), growing pigs (*n* = 60), and fattening pigs (*n* = 61) in the production stages and pregnant sows (*n* = 33). The samples were enriched in Preston broth (Oxoid Ltd., Basingstoke, United Kingdom) at 42°C for 24 h in microaerobic conditions (6% O_2_, 7.1% CO_2_, 3.6% H_2_, and 83.3% N_2_). Then, aliquots (100 μL) of the enriched broth were spread and streaked on modified charcoal-cefoperazone-deoxycholate agar plates and incubated at 42°C for 24 h in microaerobic conditions. Following incubation, 3–5 presumptive *Campylobacter* colonies per plate were selected, and DNA was extracted. *C. coli* was identified using polymerase chain reaction (PCR) with primers targeting *Campylobacter* 16S rDNA and *ask* genes (Supplementary Table 2).

### Analyzing the Aerotolerance Levels of *C. coli* Isolates

Aerotolerance levels of the *C. coli* isolates were investigated following previous methods (Oh et al., 2015; Guk et al., 2019). The isolates were cultured on Mueller-Hinton (MH) agar at 42°C for 24 h in microaerobic conditions and resuspended in fresh MH broth to a McFarland scale of 1.0 (3 × 10^8^ CFU/mL). Then, the bacterial suspension was cultured aerobically at 42°C with shaking at 200 rpm. Serial dilutions of the suspension were prepared, and inoculation on MH agar was performed at 0, 12, and 24 h after exposure to aerobic shaking. The inoculation concentrations of the suspension ranged from 10^0^ to 10^7^ dilutions, and 5 μL of each dilution was inoculated on the plates. *C. coli* isolates that lost their viability within 12 h of aerobic shaking were determined to be oxygen-sensitive (OS) strains, whereas the isolates that maintained their viability for 12–24 h were determined as AT strains. Those that remained viable for more than 24 h were considered HAT strains. Experiments were conducted in triplicate.

### Quinolone Resistance of *C. coli* Isolates

The quinolone resistance of *C. coli* isolates was tested against nalidixic acid (NAL) and ciprofloxacin (CIP). Minimum inhibitory concentration (MIC) values of the two antibiotics were determined using the broth dilution method with a Sensititre CAMPY2 plate (TREK Diagnostic Systems, Cleveland, OH, United States) and cation-adjusted Müller Hinton broth with TES (CAMHBT, TREK Diagnostic Systems). The NAL and CIP in the plate were diluted twofold with a range of 4–64 and 0.015–64 μg/mL, respectively. *C. coli* isolates were cultured on MH agar at 42°C for 24 h in microaerobic conditions, and then suspended in the cation-adjusted MH broth with TES to a McFarland standard of 0.5 (1.5 × 10^8^ CFU/mL). The suspension (100 μL) was added to a mixture of cation-adjusted MH broth with TES and 5% laked horse blood and mixed well. Then, 100 μL of the new suspension was inoculated into each well of the CAMPY2 plate containing different concentrations of the quinolone antibiotics. After the inoculation, the plates were cultured at 42°C for 24 h in microaerobic conditions. Resistance to the antibiotics was determined according to the National Antimicrobial Resistance Monitoring System (NARMS, 2019). Furthermore, high-level resistance to NAL (MIC ≥ 64 μg/mL) and CIP (MIC ≥ 32 μg/mL) was investigated by previously published methods (Segreti et al., 1992; Engberg et al., 2001). In this experiment, *C. jejuni* ATCC 33560 was used for quality control.

### Virulence Potential of *C. coli* Isolates

The prevalence of nine virulence-related genes in *C. coli* isolates was investigated using PCR. The primers and PCR conditions used in this study are presented in Supplementary Table 2. The virulence-related genes selected in this study were as follows: *flaA*, involved in motility; *cadF*, in cell adhesion; *pldA*, in colonization; *iamA*, in invasion; *ceuE*, in iron uptake system; *cdtA*, in cytotoxin production; *wlaN*, in expression of Guillain–Barré syndrome; *hcp*, in the type VI secretion system (T6SS); and *virB11*, in the type IV secretion system (T4SS) (Bacon et al., 2000; Ziprin et al., 2001; Datta et al., 2003; Bolton, 2015; Corcionivoschi et al., 2015; Koolman et al., 2015).

### Analyzing the Clonal Distribution of *C. coli* Isolates Using the Allelic Profiles of MLST Genes

The MLST genotypes of the *C. coli* isolates were determined according to the PubMLST protocol^1^. In brief, the sequences of the housekeeping genes (*aspA*, *glnA*, *gltA*, *glyA*, *pgm*, *tkt*, and *uncA*) were submitted to PubMLST, and an allele was assigned for each housekeeping gene. MLST STs were defined by using the allelic profiles of the seven housekeeping genes.

Cluster analysis of *C. coli* isolates by MLST STs was performed using the minimum spanning tree (MST) method based on the allelic profiles of the housekeeping genes with BioNumerics version 6.6 (Applied Maths, Sint-Martens-Latem, Belgium). A dendrogram was generated using the unweighted-pair group method with arithmetic mean (UPGMA) using the BioNumerics software, based on the allelic profiles of the MLST housekeeping genes for characteristics, including aerotolerance and virulence potential, of the *C. coli* isolates belonging to each MLST ST.

### Genetic Relatedness of Swine-Derived *C. coli* Isolates With Human *C. coli* Isolates

MLST data of human *C. coli* isolates were obtained from the PubMLST database and used to analyze the genetic relatedness between *C. coli* from swine and humans.

MLST data of 3,545 human *C. coli* isolates belonging to 809 MLST STs were obtained from PubMLST (accessed on 27 January 2021). Of these, 43 MLST STs with more than 10 *C. coli* isolates per ST were selected as representative MLST genotypes of human-derived *C. coli* isolates (PubMLST). The proportion of swine-derived *C. coli* isolates that shared MLST genotypes with human-derived *C. coli* was calculated and compared according to the four groups of swine and to the isolates’ aerotolerance levels.

Based on the allelic profiles of the MLST housekeeping genes, cluster analysis of *C. coli* isolates from swine and humans was conducted in BioNumerics version 6.6, following the MST method to illustrate the genetic relatedness of *C. coli* from swine and humans.

### Statistical Analysis

The prevalence of *C. coli* and the distribution of aerotolerance levels in *C. coli* were calculated with 95% confidence interval (95% CI) considering swine farms as a hierarchical level. In addition, a Generalized linear mixed-effects regression model (GLMM) with the swine farms as a random effect was applied to determine whether the prevalence of *C. coli* differed according to the swine groups using the lme4 package in the RStudio software version 1.4.1103 (Bates et al., 2014; RStudio Team, 2021). Using the same method, genetic relatedness between swine-derived *C. coli* and human *C. coli* isolates (PubMLST) was analyzed according to the swine groups and the aerotolerance levels based on MLST genotypes. Additionally, the same statistical method was used to verify whether there was a difference in characteristics, including the proportion of high-level CIP resistance and virulence-related genes, between the swine groups or between the aerotolerance levels. In the GLMM with the swine farms as a random effect, the *p*-value of 0.1 was used as the significance level.

## Results

### Prevalence and Aerotolerance Levels of *C. coli* Isolated in Different Swine Groups

A total of 124 (66.3%, 95% CI 52.9–80.8%) *C. coli* isolates were obtained from 187 swine fecal samples, and the prevalence of *C. coli* in each swine group was: 45.5% (15/33, 95% CI 22.4–67.6%) in the weaning pigs, 68.3% (41/60, 95% CI 51.2–88.3%) in the growing pigs, 75.4% (46/61, 95% CI 60.5–90.8%) in the fattening pigs, and 66.7% (22/33, 95% CI 49.9–85.6%) in the pregnant sows, showing that *C. coli* was more predominant in the fattening pigs than in the other groups. However, *C. coli* prevalence was not statistically different between the swine groups. The prevalence of *C. coli* according to the swine groups at each swine farm is in Supplementary Table 1.

We categorized the isolates according to the aerotolerance levels and determined that approximately 58.1% (*n* = 72), 28.2% (*n* = 35), and 13.7% (*n* = 17) of the 124 *C. coli* isolates were OS, AT, and HAT, respectively. Information on the aerotolerance levels of *C. coli* isolated at each swine group is presented in Table 1. As shown, the proportion of HAT *C. coli* was the highest in the pregnant sows (27.3%, 6/22) and the lowest in the weaning pigs (6.7%, 1/15). Information on the distribution of aerotolerance levels in *C. coli* isolates from swine feces at farm levels is provided in Supplementary Table 3.

**TABLE 1:** Prevalence of *Campylobacter coli* isolates according to the aerotolerance levels in each swine group.

Swine groups	No. of *C. coli* isolates (%)* (95% CI)^††^	No. of *C. coli* isolates at each Aerotolerance level**(%)^†^ (95% CI)^††^		
		OS	AT	HAT
Weaning pigs (*n* = 33)	15 (45.5%) (22.4–67.6%)	9 (60.0%) (25.1–78.9%)	5 (33.3%) (15.3–67.3%)	1 (6.7%) (−4.0–17.3%)
Growing pigs (*n* = 60)	41 (68.3%) (51.2–88.3%)	25 (61.0%) (41.6–72.3%)	11 (26.8%) (18.1–39.8%)	5 (12.2%) (3.8–24.4%)
Fattening pigs (*n* = 61)	46 (75.4%) (60.5–90.8%)	28 (60.9%) (49.6–72.2%)	13 (28.3%) (21.3–36.0%)	5 (10.9%) (4.0–16.8%)
Pregnant sows (*n* = 33)	22 (66.7%) (49.9–85.6%)	10 (45.5%) (16.9–79.8%)	6 (27.3%) (−0.3–60.3%)	6 (27.3%) (1.8–41.5%)
Total	124 (66.3%) (52.9–80.8%)	72 (58.1%) (46.3–65.5%)	35 (28.2%) (23.4–35.8%)	17 (13.7%) (10.1–18.9%)

*The prevalence of C. coli isolates at each swine group.

**OS, oxygen-sensitive; AT, aerotolerant; HAT, hyper-aerotolerant.

*^†^The proportions of C. coli isolates according to the aerotolerance levels in each swine group.*

*^††^95% CI, 95% confidence interval calculated considering the swine farms as a hierarchical level.*

### Quinolone Resistance of *C. coli* Isolates From Swine

Resistance of the *C. coli* isolates to quinolone antibiotics, including NAL and CIP, was investigated using the broth dilution method. Among the 124 *C. coli* isolates, four did not grow in the MIC tests, whereas the remaining 120 isolates exhibited resistance to both antibiotics, irrespective of the swine groups and aerotolerance levels (Supplementary Figure 1). In addition, 12 (10%) and 118 (98.3%) *C. coli* isolates showed high-level resistance to CIP (MIC ≥ 32 μg/mL) and NAL (MIC ≥ 64 μg/mL), respectively (Table 2). The proportion of *C. coli* isolates showing high-level CIP resistance at each group was: 21.4% (3/14) in the weaning pigs, 12.8% (5/39) in the growing pigs, 8.9% (4/45) in the fattening pigs, and 0% (0/22) in the pregnant sows. In terms of aerotolerance levels, the proportion of *C. coli* isolates with high-level CIP resistance was the highest in HAT *C. coli* (18.8%, 3/16), followed by AT (8.8%, 3/34) and OS (8.6%, 6/70) *C. coli*. Statistical differences were not found in the proportion of *C. coli* isolates showing high-level CIP resistance according to the swine groups and according to the aerotolerance levels.

**TABLE 2:**
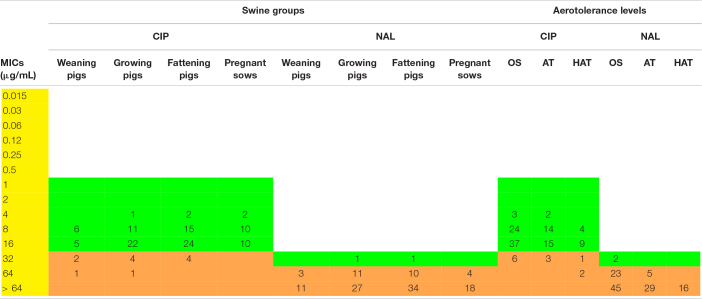
Distribution of minimum inhibitory concentration (MIC) values (μg/mL) of *Campylobacter coli* isolates according to the swine groups and aerotolerance levels.

*Green, MIC values for resistance to each antibiotic agent; Orange, MIC values for high-level resistance to each antibiotic agent; OS, oxygen-sensitive; AT, aerotolerance; HAT, hyper-aerotolerance; CIP, ciprofloxacin; NAL, nalidixic acid. Range of MIC values that were tested for CIP and NAL was 0.015–64 and 4–64 μg/mL, respectively.*

### Virulence Potential of *C. coli* Isolates From Swine

The virulence potential of *C. coli* isolates was investigated using nine major virulence-related genes. Of these, *flaA* (93.5%, 116/124), *cadF* (99.2%, 123/124), *pldA* (97.6%, 121/124), *ceuE* (93.5%, 116/124), and *cdtA* (100%, 124/124) genes were predominant (Table 3). The prevalence of virulence-related genes according to the swine groups was generally similar, except for some genes. However, for the *iamA* gene, significant differences in its prevalence according to the swine groups were identified (GLMM, *p* = 0.035, variance of the random effect = 0.432). The prevalence of the *iamA* gene was significantly higher in the fattening pigs than in the weaning pigs (fattening pigs vs. weaning pigs; GLMM, OR = 3.96; 95% CI 0.92–17.03, *p* = 0.064). In addition, the prevalence of the *ceuE* gene was different according to the swine groups (GLMM, *p* = 0.056, variance of the random effect = 0.855). The *ceuE* gene was more prevalent in the growing pigs than in the weaning pigs (growing pigs vs. weaning pigs; GLMM, OR = 9.49; 95% CI 0.84–107.33, *p* = 0.069). The *hcp* gene showed a difference in its distribution according to the swine groups (GLMM, *p* = 0.012, variance of the random effect = 0.915), and its prevalence was significantly higher in the pregnant sows than in the weaning pigs (pregnant sows vs. weaning pigs; GLMM, OR = 7.72; 95% CI 1.24–47.94, *p* = 0.028). In addition, the prevalence of virulence-related genes was generally similar according to the aerotolerance levels of *C. coli*. However, the prevalence of virulence-related genes, except for the *hcp* gene, was the highest in HAT *C. coli*. All HAT *C. coli* isolates possessed diverse virulence-related genes including *flaA*, *cadF*, *pldA*, *ceuE*, and *cdtA* genes.

**TABLE 3:** Prevalence of virulence genes of *Campylobacter coli* isolates from swine sources according to the swine groups and aerotolerance levels.

	Number of isolates	Virulence genes								
		*flaA*	*cadF*	*pldA*	*iamA*	*ceuE*	*cdtA*	*wlaN*	*hcp*	*virB11*
**Swine groups**										
Weaning pigs	15	73.3%	93.3%	86.7%	20.0%	80.0%	100%	0.0%	13.3%	6.7%
Growing pigs	41	100%	100%	100%	36.6%	97.6%	100%	2.4%	24.4%	14.6%
Fattening pigs	46	95.7%	100%	100%	47.8%	93.5%	100%	2.2%	34.8%	13.0%
Pregnant sows	22	90.9%	100%	95.5%	45.5%	95.5%	100%	9.1%	45.5%	0.0%
Aerotolerance levels*										
OS	72	91.7%	98.6%	97.2%	37.5%	90.3%	100.0%	2.8%	40.3%	9.7%
AT	35	94.3%	100.0%	97.1%	42.9%	97.1%	100.0%	2.9%	14.3%	11.4%
HAT	17	100.0%	100.0%	100.0%	47.1%	100.0%	100.0%	5.9%	23.5%	11.8%
Total	124	93.5%	99.2%	97.6%	40.3%	93.5%	100.0%	3.2%	30.6%	10.5%

*OS, oxygen-sensitive; AT, aerotolerant; HAT, hyper-aerotolerant.

### Clonal Distribution of *C. coli* Isolates Using the Allelic Profiles of Housekeeping Genes

**FIGURE 1:**
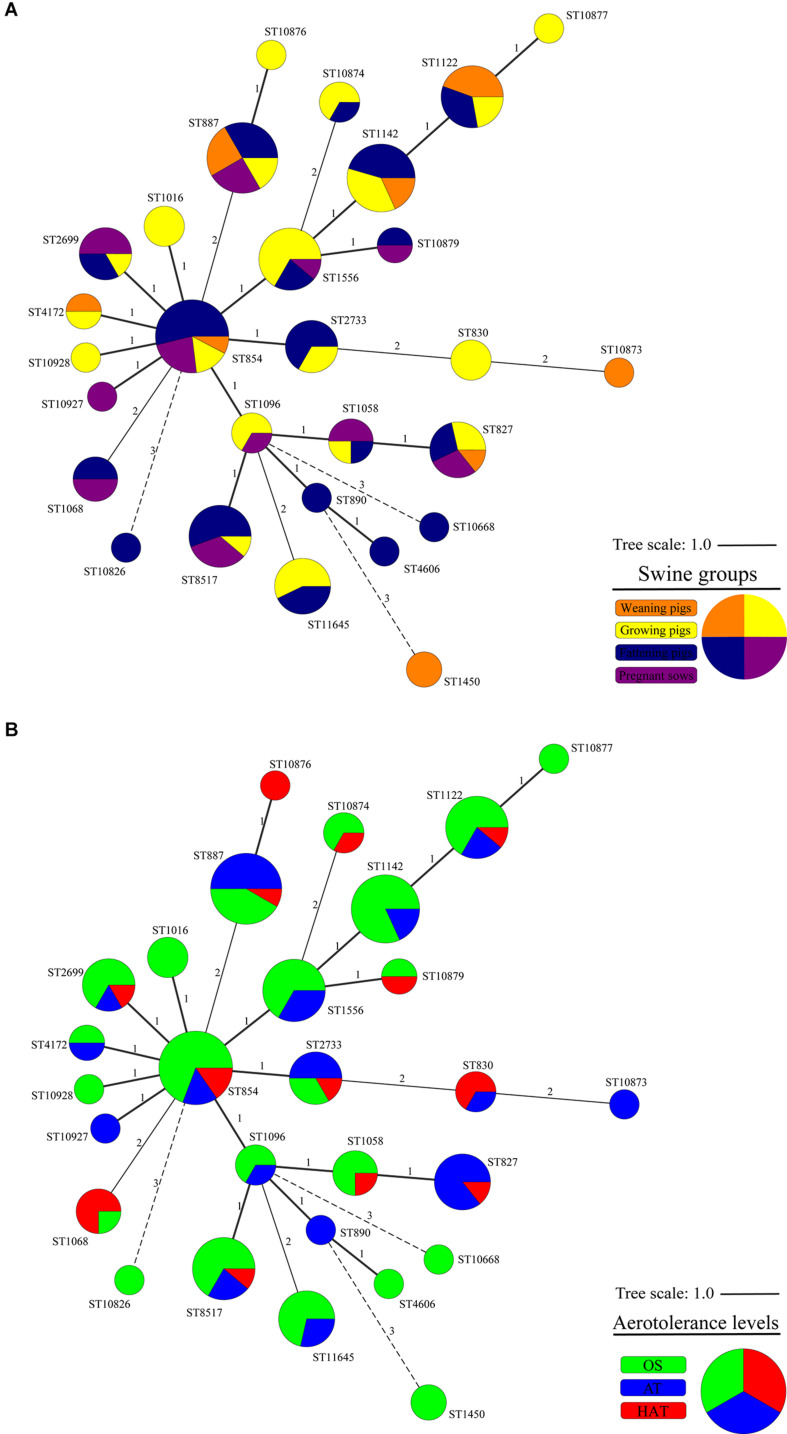
Cluster analysis of 124 *Campylobacter coli* isolates by multilocus sequence typing (MLST) sequence types (STs) according to **(A)** the swine groups and **(B)** the aerotolerance levels of *C. coli* isolates. A minimum spanning tree (MST) was constructed based on the allelic profiles of MLST housekeeping genes for 124 *C. coli* isolates. Each node represents the MLST ST, and the size of each node indicates the number of isolates belonging to each MLST ST. Colors indicate **(A)** the swine groups from which *C. coli* isolates were isolated (orange, weaning pigs; yellow, growing pigs; navy, fattening pigs; purple, pregnant sows) and **(B)** aerotolerance levels of *C. coli* (green, oxygen-sensitive, OS; blue, aerotolerant, AT; and red, hyper-aerotolerant, HAT). The numbers on the branches present the degree of difference in allelic profiles for housekeeping genes.

**FIGURE 2:**
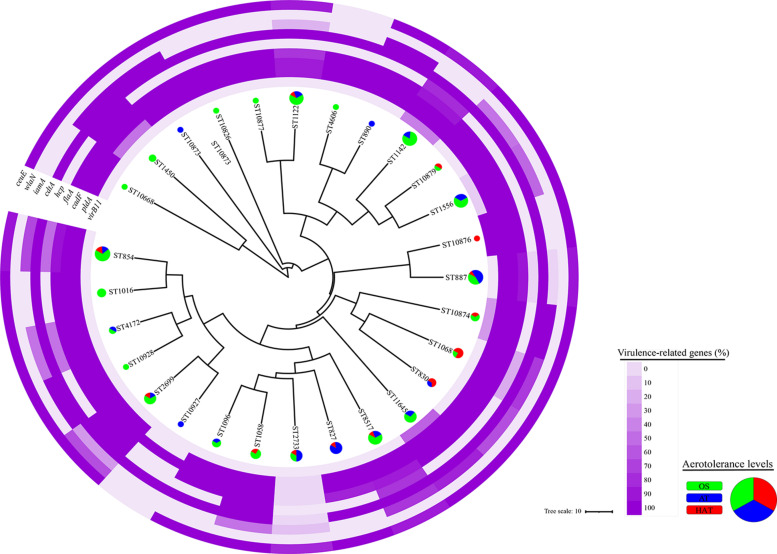
Clonal distribution of 124 *Campylobacter coli* isolates based on the allelic profiles for the multilocus sequence typing (MLST) housekeeping genes. An unweighted-pair group method with arithmetic mean (UPGMA) dendrogram was constructed based on the allelic profiles of the MLST housekeeping genes for 124 *C. coli* isolates. The UPGMA dendrogram shows the distribution of the aerotolerance levels and proportions of nine virulence genes of *C. coli* isolates in each MLST sequence type. Each color in the pie chart indicates an aerotolerance level (green, oxygen-sensitive, OS; blue, aerotolerant, AT; and red, hyper-aerotolerant, HAT) of *C. coli* isolates.

From the 124 swine-derived *C*. *coli* isolates, 28 MLST STs were identified. Generally, all isolates were closely clustered, regardless of the swine groups and aerotolerance levels (Figure 1). Most (87.9%, 109/124) *C. coli* strains belonged to the same MLST clonal complex (CC-828), except for *C. coli*, whose MLST CCs had not been determined yet. In addition, three main MLST genotypes (ST827, ST854, ST887) that covered all swine groups were identified in 32 *C. coli* isolates (25.8%) (Figure 1A). The distribution of MLST genotypes in *C. coli* isolates from swine feces at farm levels is provided in Supplementary Table 4. The UPGMA dendrogram showed that most of the isolates possessed various virulence-related genes, regardless of the MLST genotype (Figure 2). Information on the MLST genotype of each *C. coli* isolate is listed in Supplementary Table 5.

### Genetic Relatedness of Swine-Derived *C. coli* Isolates With Human *C. coli* Isolates

**FIGURE 3:**
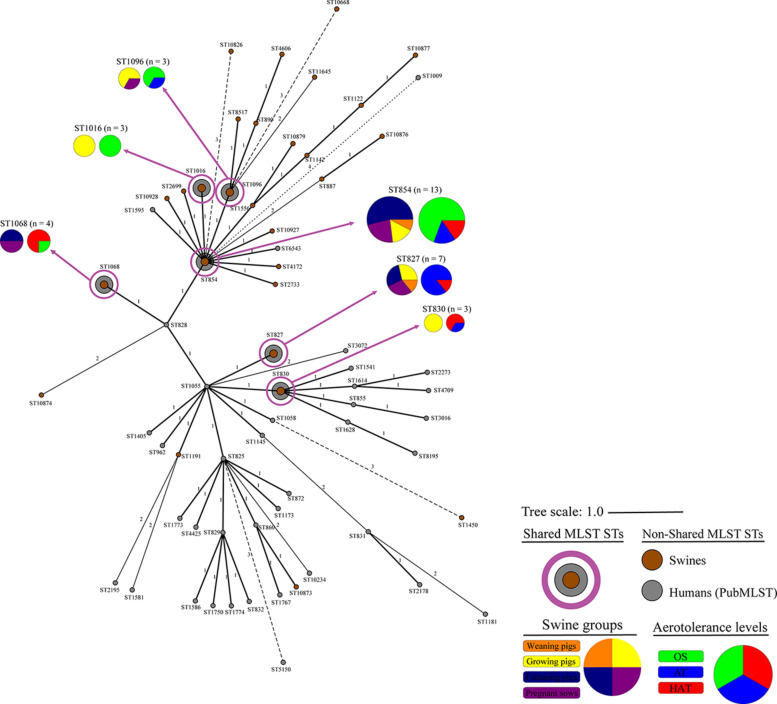
Genetic relatedness of swine-derived *Campylobacter coli* isolates in this study with human *C. coli* isolates registered in the PubMLST. Cluster analysis of *C. coli* isolates from swine and humans was conducted using the MST method, based on the allelic profiles of the MLST housekeeping genes. Colors indicate the sources (brown, swine from this study; and gray, humans from PubMLST), the swine groups (orange, weaning pigs; yellow, growing pigs; navy, fattening pigs; and purple, pregnant sows), and aerotolerance levels of *C. coli* isolates (green, oxygen-sensitive, OS; blue, aerotolerant, AT; and red, hyper-aerotolerant, HAT). The numbers on the branches present the difference degree in allelic profiles for housekeeping genes.

Forty-three MLST STs, among the 809 MLST genotypes obtained from the PubMLST database, containing 2,314 *C. coli* isolates, were selected as representative genotypes of human *C. coli* isolates and used in the analysis of genetic relatedness with swine-derived *C*. *coli* isolates. Six of the 28 MLST STs from swine (ST827, ST830, ST854, ST1016, ST1068, ST1096) were shared with humans (Figure 3), including approximately 26.6% (33/124) and 35.6% (823/2314) of *C. coli* isolates from swine and humans, respectively.

The proportion of *C. coli* belonging to the six shared STs at each swine group was the highest in the pregnant sows (36.4%, 8/22) (Table 4). In addition, the proportion at each aerotolerance level was the highest in HAT *C. coli* (47.1%, 8/17), followed by AT (28.6%, 10/35) and OS *C. coli* (20.8%, 15/72) (Table 4). The genetic relatedness between swine-derived *C. coli* and human *C. coli* isolates according to the swine groups was statistically different between the swine groups (GLMM, *p* = 0.014, variance of the random effect = 0.605). The proportion of *C. coli* in the shared STs was significantly higher in the pregnant sows than in the weaning pigs (pregnant sows vs. weaning pigs; GLMM, OR = 5.55; 95% CI 0.85–36.39, *p* = 0.074). Furthermore, the genetic relatedness between swine-derived *C. coli* and human *C. coli* isolates according to the aerotolerance levels was significantly different (GLMM, *p* = 0.001, variance of the random effect = 0.343). The proportion of *C. coli* in the shared STs in HAT *C. coli* was significantly higher than in OS *C. coli* (HAT vs. OS; GLMM, OR = 3.13; 95% CI 0.99–9.96, *p* = 0.053) (Table 4).

**TABLE 4:** *Campylobacter coli* in the shared multilocus sequence typing (MLST) sequence types (STs) with human *C. coli* isolates (PubMLST) according to the swine groups and aerotolerance levels of *C. coli*.

	Shared STs*	Non-shared STs**	OR (95% CI) and *p*-values^††^ (GLMM)	
**Swine groups**				*p* = 0.014**^†^**
Weaning pigs (*n* = 15)	2 (13.3%)	13 (86.7%)	−	
Growing pigs (*n* = 41)	12 (29.3%)	29 (70.7%)	3.97 (0.67–23.43)	*p* = 0.129
Fattening pigs (*n* = 46)	11 (23.9%)	35 (76.1%)	2.74 (0.47–15.83)	*p* = 0.261
Pregnant sows (*n* = 22)	8 (36.4%)	14 (63.6%)	5.55 (0.85–36.39)	*p* = 0.074
**Aerotolerance levels**				*p* = 0.001**^†^**
Oxygen-sensitive (OS) (*n* = 72)	15 (20.8%)	57 (79.2%)	−	
Aerotolerant (AT) (*n* = 35)	10 (28.6%)	25 (71.4%)	1.34 (0.51–3.54)	*p* = 0.556
Hyper-aerotolerant (HAT) (*n* = 17)	8 (47.1%)	9 (52.9%)	3.13 (0.99–9.96)	*p* = 0.053
Total	33 (26.6%)	91 (73.4%)		
	124			

*Shared STs; six MLST sequence types (ST827, ST830, ST854, ST1016, ST1068, and ST1096) shared between swine and humans (PubMLST).

**Non-shared STs; MLST STs not shared between swine and humans (PubMLST).

*^†^Global p-values; p-values from the analysis of the genetic relatedness between swine-derived C. coli and human C. coli isolates according to the swine groups and aerotolerance levels, respectively, using a GLMM (Generalized linear mixed-effects regression model) with swine farms as a random effect.*

*^††^OR (95% confidence interval, CI) and p-values; odds ratio (95% CI). The OR and p-values were calculated for the swine groups and aerotolerance levels using the weaning pigs and the oxygen-sensitive (OS) strains as a reference, respectively.*

## Discussion

The aerotolerance of *Campylobacter* has been particularly concerning recently because aerotolerant strains are prevalent in poultry, and these strains are closely related to human *Campylobacter* infections (Oh et al., 2015; Karki et al., 2018; Guk et al., 2019; Song et al., 2020). However, the aerotolerance of *C. coli* isolated from pigs, another important host of *C. coli*, has not been studied yet (Kempf et al., 2017; Riedel et al., 2020). In this study, we investigated the prevalence of *C. coli* in six pig farms according to four swine groups including pigs in three production stages (weaning, growing, fattening) and pregnant sows. *Campylobacter coli* characteristics, including aerotolerance, quinolone resistance, virulence, and MLST genotype, were compared for each swine group. In addition, we evaluated the potential impacts of swine-derived HAT *C. coli* on food safety by analyzing quinolone resistance, virulence potential, and genetic relatedness of *C. coli* according to aerotolerance levels. This is the first study to compare the prevalence and characteristics of swine-derived *C. coli* according to the swine groups and aerotolerance levels.

We identified that the majority of swine fecal samples were positive for *C. coli* (66.3%, 124/187, 95% CI 52.9–80.8%). The prevalence of *C. coli* in pigs in this study was higher than that in other livestock, including cattle (7.4–15.0%) and poultry, such as chickens (26.4–40.2%) and ducks (46.6–57.9%) (Bae et al., 2005; Kang et al., 2006; Little et al., 2008; Torralbo et al., 2015; Tang et al., 2017; Guk et al., 2019; Karama et al., 2020). This was similar to the results of a previous study reporting a high prevalence (56–77%) of *C. coli* in swine colon samples (Kempf et al., 2017), indicating that pigs are a major reservoir of *C. coli*. The prevalence of *C. coli* according to the swine groups was the highest in the fattening pigs (75.4%). Pigs are grown in swine farms until the fattening stage and then shipped to slaughterhouses. Therefore, this high prevalence of *C. coli* in the fattening pigs suggests that *C. coli* might be spread out of the farms when fattening pigs are sent to slaughterhouses. In addition, it suggests the possibility of *C. coli* transmissions to humans during the farm-to-table process.

HAT *C. coli* was less prevalent (13.7%) in pigs compared with OS and AT *C. coli*. This finding was contrary to previous studies reporting high proportions (49.1–50%) of HAT *C. coli* in poultry, including chicken livers and duck sources (Karki et al., 2018; Guk et al., 2019). However, we identified that the proportion of HAT *C. coli* was the highest in pregnant sows. This suggests that HAT *C. coli* is likely to remain in pig farms and re-infect other pigs in the same farms through fecal-to-oral transmission, considering that pregnant sows reside in the pig farms. Further studies on transmission dynamics of HAT *C. coli* in pig farms are required to confirm if re-infection in these farms is plausible.

Quinolone resistance in *Campylobacter* and the incidence of *Campylobacter* infections in humans have been dramatically increasing worldwide (Engberg et al., 2001; Moore et al., 2006; Alfredson and Korolik, 2007; Luangtongkum et al., 2009). Indeed, the occurrence of resistance to CIP in *Campylobacter* from humans was in a range from high to extremely high according to a previous report by the European Food Safety Authority and European Centre for Disease Prevention and Control (2021). Moreover, *Campylobacter* was listed as one of the high-priority antimicrobial-resistant pathogens by World Health Organization owing to the increase of fluoroquinolone resistance, and fluoroquinolone-resistant *Campylobacter* was identified as a serious public health threat by the Centers for Disease Control and Prevention (World Health Organization, 2017; Centers for Disease Control and Prevention, 2019). In this study, all strains, except for four *C. coli* strains that did not grow in the MIC tests, showed resistance to both CIP and NAL, suggesting that resistance to quinolones may be distributed in *C. coli* in swine throughout the swine groups, irrespective of the aerotolerance levels. Furthermore, the proportion of high-level CIP-resistant *C. coli* was the highest in HAT *C. coli* (18.8%, 3/16). These findings indicate that quinolone-resistant *C. coli* could be transmitted to humans from swine, potentially making it difficult to treat human *Campylobacter* infections because quinolones are used in campylobacteriosis therapy (Sifré et al., 2015; Tang et al., 2017; Guk et al., 2019). Moreover, it could be even more difficult to treat patients infected with HAT *C. coli* strains because these strains tend to be highly resistant to fluoroquinolones such as CIP.

Most virulence-related genes in this study were distributed similarly in swine-derived *C. coli* strains, irrespective of the swine groups and the aerotolerance levels. Additionally, all HAT *C. coli* strains possessed various major virulence-related genes which are associated with motility, cell adhesion, colonization, iron uptake, and toxin production (Ziprin et al., 2001; Bolton, 2015). This suggests that the HAT *C. coli* from pigs might be more pathogenic than other *C. coli* strains owing to their high-level CIP resistance and virulence. This finding is similar to that of our previous study that reported a high prevalence (75–96.4%) of diverse virulence-related genes in HAT *C. coli* strains from ducks (Guk et al., 2019). Similarly, there was no significant difference in the proportions of virulence-related genes between HAT strains and other strains, as seen in *C. coli* isolated from ducks (Guk et al., 2019). However, a previous study on *C. jejuni* from chickens reported that the prevalence of several virulence-related genes was significantly higher in HAT *C. jejuni* than in OS *C. jejuni* (Oh et al., 2017). This could be attributed to the species differences between *C. coli* and *C. jejuni* or to differences in adaptation to hosts. However, further studies on interspecies differences in *Campylobacter* species or their adaptations in host environments are needed.

All *C. coli* strains were closely clustered regardless of the swine groups and the aerotolerance levels. Of the 32 strains belonging to the three major MLST STs (ST827, ST854, and ST887) that covered all swine groups, most (62.5%) *C. coli* belonged to ST827 and ST854, which were identified as major STs in the human isolates (PubMLST) and accounted for 32.5% of human *C. coli* isolates. These findings indicate that *C. coli* belonging to certain MLST genotypes may circulate throughout all swine groups in pig farms by horizontal or vertical transmission. Moreover, these results imply the possibility that *C. coli*—which may be genetically related to human *C. coli* isolates—is likely to be present in pig farms, indicating the possible transmission of *C. coli* from swine to humans. Furthermore, HAT *C. coli* strains from swine constituted a considerable proportion of the shared STs (ST827, ST830, ST854, ST1016, ST1068, and ST1096) with human *C. coli* isolates compared with OS *C. coli* strains, indicating a potential high genetic relatedness between swine-derived HAT *C. coli* and human *C. coli* isolates. This was consistent with the results of our previous study on aerotolerance of *C. coli* in ducks; we found that HAT *C. coli* from ducks accounted for a higher proportion of the shared MLST genotypes with the human *C. coli* isolates (PubMLST) compared with OS *C. coli* (Guk et al., 2019). Taken together, it can be speculated that the aerotolerance of *C. coli* might affect *Campylobacter* infections in humans, and that HAT *C. coli* might be transmitted to humans through the food chain. Our speculation is supported by a previous study, which revealed that most *C. jejuni* strains from human clinical cases were HAT (Oh et al., 2018).

In conclusion, resistance to quinolones may be distributed in our *C. coli* isolates in swine throughout all swine groups. In addition, HAT *C. coli* was predominant in the pregnant sows, suggesting that HAT *C. coli* is likely to remain in pig farms and might re-infect other pigs in the same farms. All HAT *C. coli* strains, including some of them showing high-level CIP resistance, showed quinolone resistance and harbored various virulence-related genes associated with motility, cell adhesion, colonization, iron uptake, and toxin production. These findings imply that swine-derived HAT *C. coli* could be a potential threat to public health owing to their high-level quinolone resistance and virulence. Furthermore, HAT *C. coli* from swine constituted a highest proportion of the shared MLST genotypes with human isolates (PubMLST), indicating that HAT *C. coli* might be transmitted to humans through the food chain given its aerotolerance. Further studies are needed to elucidate the mechanisms underlying the transmission of swine-derived HAT *C. coli* to humans through the farm-to-table process. This study highlights the need to improve management practices to diminish the transmission of swine-derived HAT *C. coli* to humans.

## Data Availability Statement

The original contributions presented in the study are included in the article/Supplementary Material, further inquiries can be directed to the corresponding author/s.

## Author Contributions

SC and W-HK conceived and designed the study. HS, SY, J-UA, SL, and J-HG performed the sampling and experiments. J-HG analyzed the data and made a great contribution to the experiments, data analysis, and preparing the manuscript. SC, W-HK, and J-HG prepared and reviewed the manuscript. All authors contributed to the article and approved the submitted version.

## Conflict of Interest

The authors declare that the research was conducted in the absence of any commercial or financial relationships that could be construed as a potential conflict of interest.

## Footnotes

1
http://pubmlst.org/campylobacter
