# Supplementary material for: Hyper-Aerotolerant Campylobacter coli From Swine May Pose a Potential Threat to Public Health Based on Its Quinolone Resistance, Virulence Potential, and Genetic Relatedness
Source: Front Microbiol. 2021 Jul 16;12:703993. doi: 10.3389/fmicb.2021.703993 (PMC8352582; doi:10.3389/fmicb.2021.703993)
Supplement: Supplementary file 1 [file Data_Sheet_1.docx]

Supplementary Material

Supplementary Figure 1. Minimum inhibitory concentration (MIC) values of (A) nalidixic acid and (B) ciprofloxacin in *Campylobacter coli* isolates arranged according to aerotolerance levels at each swine group. MIC values are expressed as log_2_ MICs (μg/mL). Dotted lines indicate break points, expressed as the log value, for resistance to each antibiotic in *C. coli* isolates. Dashed lines indicate break points, expressed as the log value, for high-level resistance to each antibiotic in *C. coli* isolates. OS, oxygen-sensitive (green); AT, aerotolerant (blue); HAT, hyper-aerotolerant (red).


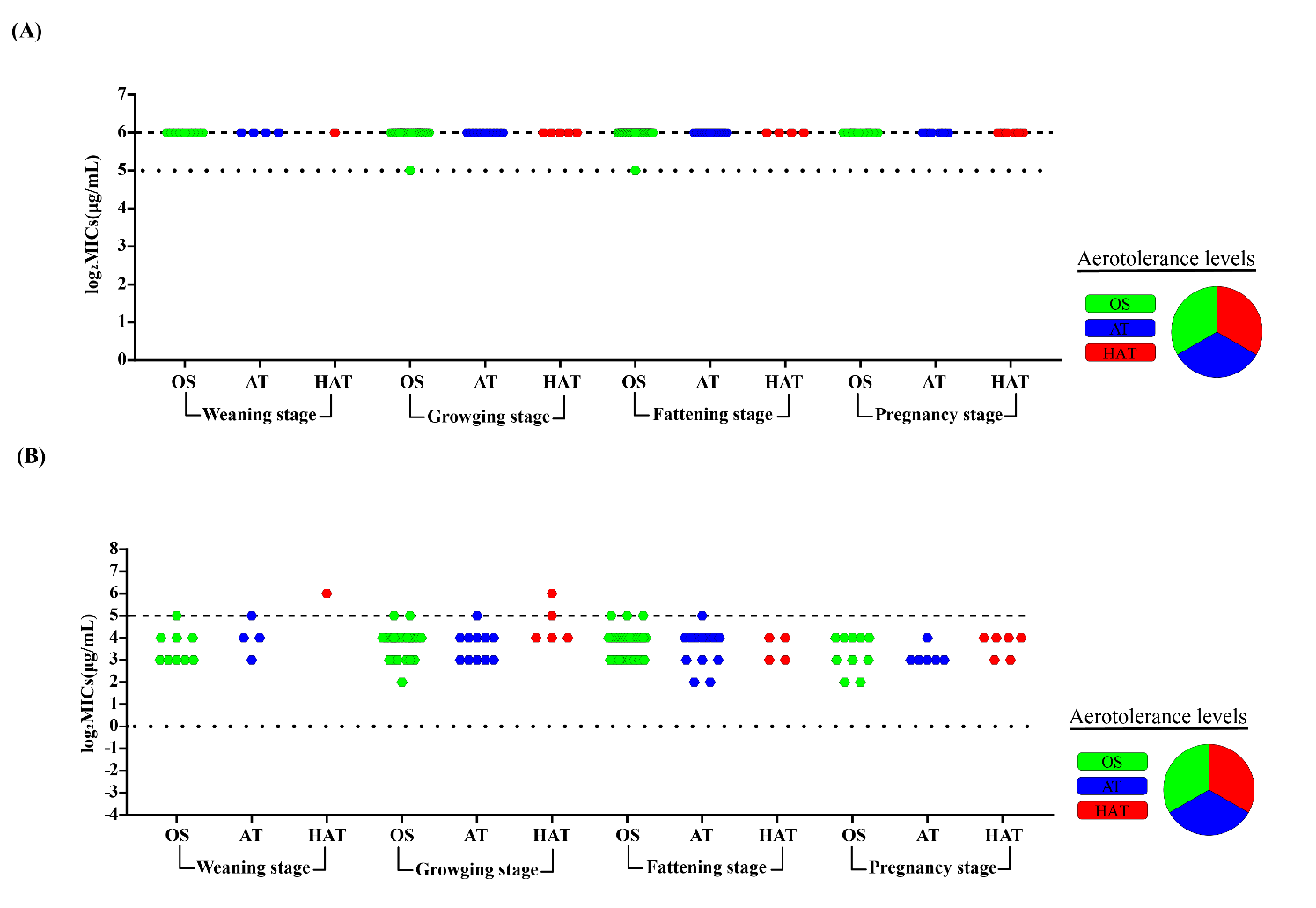


Supplementary Table 1. Prevalence of *Campylobacter coli* according to the swine groups at six swine farms.

| **Swine groups** | **Farm 1** | **Farm 2** | **Farm 3** | **Farm 4** | **Farm 5** | **Farm 6** |
| --- | --- | --- | --- | --- | --- | --- |
|  | Prevalence (Number of isolates^*^/Number of samples^**^) | Prevalence (Number of isolates/Number of samples) | Prevalence (Number of isolates/Number of samples) | Prevalence (Number of isolates/Number of samples) | Prevalence (Number of isolates/Number of samples) | Prevalence (Number of isolates/Number of samples) |
| Weaning pigs | 83.3% (5/6) | 60% (3/5) | 16.7% (1/6) | 50% (3/6) | 0% (0/5) | 60% (3/5) |
| Growing pigs | 90.9% (10/11) | 100% (8/8) | 63.6% (7/11) | 27.3% (3/11) | 66.7% (6/9) | 70% (7/10) |
| Fattening pigs | 100% (11/11) | 87.5% (7/8) | 90.9% (10/11) | 45.5% (5/11) | 60% (6/10) | 70% (7/10) |
| Pregnant sows | 83.3% (5/6) | 80% (4/5) | 50% (3/6) | 33.3% (2/6) | 100% (5/5) | 60% (3/5) |
| Total | 91.2% (31/34) | 84.6% (22/26) | 61.8% (21/34) | 38.2% (13/34) | 58.6% (17/29) | 66.7% (20/30) |

^*^The number of *C. coli* isolates from swine fecal samples at each swine group in each farm.

^**^The number of swine fecal samples obtained at each swine group in each farm.

Supplementary Table 2. Primers for identification of *Campylobacter coli* and its virulence genes.

| **Primers** | | **Primer Sequence (5'–3')** | **Annealing Temperature (°C)** | **References** |
| --- | --- | --- | --- | --- |
| *Campylobacter* 16S rDNA | F | GGA TGA CAC TTT TCG GAG C | 57 | (Linton, Owen et al. 1996) |
|  | R | CAT TGT AGC ACG TGT GTC |  |  |
| *ask* | F | GGT ATG ATT TCT ACA AAG CGA G | 57 | (Linton, Lawson et al. 1997) |
|  | R | ATA AAA GAC TAT CGT CGC GTG |  |  |
| *flaA* | F | GGATTTCGTATTAACACAAATGGTGC | 55 | (Nachamkin, Ung et al. 1996) |
|  | R | CTGTAGTAATCTTAAAACATTTTG |  |  |
| *cadF* | F | TTGAAGGTAATTTAGATATG | 45 | (Konkel, Gray et al. 1999) |
|  | R | CTAATACCTAAAGTTGAAAC |  |  |
| *pldA* | F | AAGCTTATGCGTTTTT | 45 | (Datta, Niwa et al. 2003) |
|  | R | TATAAGGCTTTCTCCA |  |  |
| *iamA* | F | GCACAAAATATATCATTACAA | 52 | (Müller, Schulze et al. 2006) |
|  | R | TTCACGACTACTATGAGG |  |  |
| *ceuE* | F | ATGAAAAAATATTTAGTTTTTGCA | 57 | (Gonzalez, Grant et al. 1997) |
|  | R | ATTTTATTATTTGTAGCAGCG |  |  |
| *cdtA* | F | GGAAATTGGATTTGGGGCTATACT | 42 | (Bang, Scheutz et al. 2001, Bang, Nielsen et al. 2003) |
|  | R | ATCACAAGGATAATGGACAAT |  |  |
| *wlaN* | F | TGCTGGGTATACAAAGGTTGTG | 60 | (Müller, Schulze et al. 2006) |
|  | R | AATTTTGGATATGGGTGGGG |  |  |
| *hcp* | F | CAAGCGGTGCATCTACTGAA | 55 | (Bleumink-Pluym, van Alphen et al. 2013, Corcionivoschi, Gundogdu et al. 2015) |
|  | R | TAAGCTTTGCCCTCTCTCCA |  |  |
| *virB11* | F | GAACAGGAAGTGGAAAAACTAGC | 50 | (Bacon, Alm et al. 2000) |
|  | R | TTCCGCATTGGGCTATATG |  |  |

Supplementary Table 3. Distribution of aerotolerance levels in *Campylobacter coli* isolates from swine feces at farm levels.

| **Aerotolerance levels^†^** | **Farm 1** | **Farm 2** | **Farm 3** | **Farm 4** | **Farm 5** | **Farm 6** |
| --- | --- | --- | --- | --- | --- | --- |
|  | Prevalence (Number of isolates^*^/Number of samples^**^) | Prevalence (Number of isolates/Number of samples) | Prevalence (Number of isolates/Number of samples) | Prevalence (Number of isolates/Number of samples) | Prevalence (Number of isolates/Number of samples) | Prevalence (Number of isolates/Number of samples) |
| OS | 55.9% (19/34) | 53.8% (14/26) | 41.2% (14/34) | 11.8% (4/34) | 31.0% (9/29) | 40.0% (12/30) |
| AT | 23.5% (8/34) | 19.2% (5/26) | 17.6% (6/34) | 17.6% (6/34) | 17.2% (5/29) | 16.7% (5/30) |
| HAT | 11.8% (4/34) | 11.5% (3/26) | 2.9% (1/34) | 8.8% (3/34) | 10.3% (3/29) | 10.0% (3/30) |
| Total | 91.2% (31/34) | 84.6% (22/26) | 61.8% (21/34) | 38.2% (13/34) | 58.6% (17/29) | 66.7% (20/30) |

^*^The number of *C. coli* isolates at each aerotolerance level in each farm.

^**^The number of swine fecal samples obtained from each farm.

**^†^**OS, oxygen-sensitive; AT, aerotolerant; HAT, hyper-aerotolerant.

Supplementary Table 4. Distribution of multilocus sequence typing (MLST) genotypes in *Campylobacter coli* isolates from swine feces at farm levels.

| **MLST STs** | **Farm 1** | **Farm 2** | **Farm 3** | **Farm 4** | **Farm 5** | **Farm 6** |
| --- | --- | --- | --- | --- | --- | --- |
|  | Prevalence (Number of isolates^*^/Number of samples^**^) | Prevalence (Number of isolates/Number of samples) | Prevalence (Number of isolates/Number of samples) | Prevalence (Number of isolates/Number of samples) | Prevalence (Number of isolates/Number of samples) | Prevalence (Number of isolates/Number of samples) |
| 827 | 0% (0/34) | 0% (0/26) | 0% (0/34) | 20.6% (7/34) | 0% (0/29) | 0% (0/30) |
| 830 | 0% (0/34) | 0% (0/26) | 0% (0/34) | 0% (0/34) | 0% (0/29) | 10% (3/30) |
| 854 | 17.6% (6/34) | 0% (0/26) | 2.9% (1/34) | 2.9% (1/34) | 3.4% (1/29) | 13.3% (4/30) |
| 887 | 8.8% (3/34) | 15.4% (4/26) | 8.8% (3/34) | 0% (0/34) | 0% (0/29) | 6.7% (2/30) |
| 890 | 0% (0/34) | 0% (0/26) | 0% (0/34) | 0% (0/34) | 3.4% (1/29) | 0% (0/30) |
| 1016 | 0% (0/34) | 0% (0/26) | 8.8% (3/34) | 0% (0/34) | 0% (0/29) | 0% (0/30) |
| 1058 | 0% (0/34) | 7.7% (2/26) | 5.9% (2/34) | 0% (0/34) | 0% (0/29) | 0% (0/30) |
| 1068 | 0% (0/34) | 0% (0/26) | 0% (0/34) | 2.9% (1/34) | 10.3% (3/29) | 0% (0/30) |
| 1096 | 0% (0/34) | 11.5% (3/26) | 0% (0/34) | 0% (0/34) | 0% (0/29) | 0% (0/30) |
| 1122 | 17.6% (6/34) | 0% (0/26) | 0% (0/34) | 0% (0/34) | 0% (0/29) | 10% (3/30) |
| 1142 | 2.9% (1/34) | 26.9% (7/26) | 2.9% (1/34) | 2.9% (1/34) | 0% (0/29) | 3.3% (1/30) |
| 1450 | 0% (0/34) | 7.7% (2/26) | 0% (0/34) | 0% (0/34) | 0% (0/29) | 0% (0/30) |
| 1556 | 2.9% (1/34) | 0% (0/26) | 0% (0/34) | 0% (0/34) | 27.6% (8/29) | 0% (0/30) |
| 2699 | 5.9% (2/34) | 3.8% (1/26) | 2.9% (1/34) | 5.9% (2/34) | 0% (0/29) | 0% (0/30) |
| 2733 | 0% (0/34) | 0% (0/26) | 8.8% (3/34) | 0% (0/34) | 0% (0/29) | 10% (3/30) |
| 4172 | 0% (0/34) | 0% (0/26) | 2.9% (1/34) | 0% (0/34) | 0% (0/29) | 3.3% (1/30) |
| 4606 | 0% (0/34) | 0% (0/26) | 0% (0/34) | 0% (0/34) | 0% (0/29) | 3.3% (1/30) |
| 8517 | 0% (0/34) | 3.8% (1/26) | 17.6% (6/34) | 2.9% (1/34) | 0% (0/29) | 3.3% (1/30) |
| 10668 | 0% (0/34) | 0% (0/26) | 0% (0/34) | 0% (0/34) | 0% (0/29) | 3.3% (1/30) |
| 10826 | 0% (0/34) | 0% (0/26) | 0% (0/34) | 0% (0/34) | 3.4% (1/29) | 0% (0/30) |
| 10873 | 2.9% (1/34) | 0% (0/26) | 0% (0/34) | 0% (0/34) | 0% (0/29) | 0% (0/30) |
| 10874 | 8.8% (3/34) | 0% (0/26) | 0% (0/34) | 0% (0/34) | 0% (0/29) | 0% (0/30) |
| 10876 | 0% (0/34) | 3.8% (1/26) | 0% (0/34) | 0% (0/34) | 0% (0/29) | 0% (0/30) |
| 10877 | 0% (0/34) | 3.8% (1/26) | 0% (0/34) | 0% (0/34) | 0% (0/29) | 0% (0/30) |
| 10879 | 0% (0/34) | 0% (0/26) | 0% (0/34) | 0% (0/34) | 6.9% (2/29) | 0% (0/30) |
| 10927 | 2.9% (1/34) | 0% (0/26) | 0% (0/34) | 0% (0/34) | 0% (0/29) | 0% (0/30) |
| 10928 | 0% (0/34) | 0% (0/26) | 0% (0/34) | 0% (0/34) | 3.4% (1/29) | 0% (0/30) |
| 11645 | 20.6% (7/34) | 0% (0/26) | 0% (0/34) | 0% (0/34) | 0% (0/29) | 0% (0/30) |
| Total | 91.2% (31/34) | 84.6% (22/26) | 61.8% (21/34) | 38.2% (13/34) | 58.6% (17/29) | 66.7% (20/30) |

^*^The number of *C. coli* isolates belonging to each MLST STs in each farm.

^**^The number of swine fecal samples obtained from each farm.

**^†^**MLST STs, MLST sequence types.

Supplementary Table 5. *Campylobacter coli* isolates from swine feces according to the swine groups.

| **Strain IDs** | **Swine groups** | **MLST STs** | **MLST CCs^*^** | **Aerotolerance levels^**^** | **Swine farms** |
| --- | --- | --- | --- | --- | --- |
| CC001 | Weaning pigs | 4172 | 828 | AT | Farm 3 |
| CC002 |  | 854 | 828 | HAT | Farm 4 |
| CC003 |  | 1142 | 828 | OS | Farm 4 |
| CC004 |  | 827 | 828 | AT | Farm 4 |
| CC005 |  | 887 | 828 | OS | Farm 6 |
| CC006 |  | 887 | 828 | OS | Farm 6 |
| CC007 |  | 1142 | 828 | AT | Farm 6 |
| CC008 |  | 10873 | ND | AT | Farm 1 |
| CC009 |  | 1122 | 828 | OS | Farm 1 |
| CC010 |  | 1122 | 828 | OS | Farm 1 |
| CC011 |  | 1122 | 828 | OS | Farm 1 |
| CC012 |  | 1122 | 828 | AT | Farm 1 |
| CC013 |  | 887 | 828 | OS | Farm 2 |
| CC014 |  | 1450 | ND | OS | Farm 2 |
| CC015 |  | 1450 | ND | OS | Farm 2 |
| CC016 | Growing pigs | 1142 | 828 | OS | Farm 3 |
| CC017 |  | 1016 | 828 | OS | Farm 3 |
| CC018 |  | 1058 | 828 | OS | Farm 3 |
| CC019 |  | 8517 | 828 | OS | Farm 3 |
| CC020 |  | 1016 | 828 | OS | Farm 3 |
| CC021 |  | 1016 | 828 | OS | Farm 3 |
| CC022 |  | 887 | 828 | AT | Farm 3 |
| CC023 |  | 827 | 828 | AT | Farm 4 |
| CC024 |  | 827 | 828 | HAT | Farm 4 |
| CC025 |  | 2699 | 828 | OS | Farm 4 |
| CC026 |  | 830 | 828 | AT | Farm 6 |
| CC027 |  | 830 | 828 | HAT | Farm 6 |
| CC028 |  | 2733 | 828 | AT | Farm 6 |
| CC029 |  | 1122 | 828 | OS | Farm 6 |
| CC030 |  | 4172 | 828 | OS | Farm 6 |
| CC031 |  | 830 | 828 | HAT | Farm 6 |
| CC032 |  | 2733 | 828 | OS | Farm 6 |
| CC033 |  | 1556 | 828 | OS | Farm 1 |
| CC034 |  | 11645 | ND | AT | Farm 1 |
| CC035 |  | 1122 | 828 | OS | Farm 1 |
| CC036 |  | 11645 | ND | OS | Farm 1 |
| CC037 |  | 10874 | 828 | OS | Farm 1 |
| CC038 |  | 11645 | ND | OS | Farm 1 |
| CC039 |  | 854 | 828 | OS | Farm 1 |
| CC040 |  | 854 | 828 | OS | Farm 1 |
| CC041 |  | 11645 | ND | OS | Farm 1 |
| CC042 |  | 10874 | 828 | HAT | Farm 1 |
| CC043 |  | 1142 | 828 | OS | Farm 2 |
| CC044 |  | 887 | 828 | AT | Farm 2 |
| CC045 |  | 1142 | 828 | AT | Farm 2 |
| CC046 |  | 10876 | ND | HAT | Farm 2 |
| CC047 |  | 1142 | 828 | OS | Farm 2 |
| CC048 |  | 1096 | 828 | AT | Farm 2 |
| CC049 |  | 1096 | 828 | OS | Farm 2 |
| CC050 |  | 1556 | 828 | AT | Farm 5 |
| CC051 |  | 1556 | 828 | AT | Farm 5 |
| CC052 |  | 1556 | 828 | AT | Farm 5 |
| CC053 |  | 1556 | 828 | OS | Farm 5 |
| CC054 |  | 1556 | 828 | OS | Farm 5 |
| CC055 |  | 10928 | 828 | OS | Farm 5 |
| CC056 |  | 10877 | ND | OS | Farm 2 |
| CC057 | Fattening pigs | 8517 | 828 | OS | Farm 3 |
| CC058 |  | 887 | 828 | AT | Farm 3 |
| CC059 |  | 2733 | 828 | OS | Farm 3 |
| CC060 |  | 854 | 828 | OS | Farm 3 |
| CC061 |  | 2733 | 828 | HAT | Farm 3 |
| CC062 |  | 8517 | 828 | OS | Farm 3 |
| CC063 |  | 2733 | 828 | AT | Farm 3 |
| CC064 |  | 1058 | 828 | OS | Farm 3 |
| CC065 |  | 8517 | 828 | AT | Farm 3 |
| CC066 |  | 8517 | 828 | AT | Farm 3 |
| CC067 |  | 827 | 828 | AT | Farm 4 |
| CC068 |  | 1068 | 828 | HAT | Farm 4 |
| CC069 |  | 8517 | 828 | OS | Farm 4 |
| CC070 |  | 827 | 828 | AT | Farm 4 |
| CC071 |  | 2699 | 828 | OS | Farm 4 |
| CC072 |  | 1122 | 828 | HAT | Farm 6 |
| CC073 |  | 2733 | 828 | AT | Farm 6 |
| CC074 |  | 4606 | ND | OS | Farm 6 |
| CC075 |  | 10668 | ND | OS | Farm 6 |
| CC076 |  | 854 | 828 | OS | Farm 6 |
| CC077 |  | 1122 | 828 | AT | Farm 6 |
| CC078 |  | 854 | 828 | OS | Farm 6 |
| CC079 |  | 10874 | 828 | OS | Farm 1 |
| CC080 |  | 887 | 828 | HAT | Farm 1 |
| CC081 |  | 11645 | ND | OS | Farm 1 |
| CC082 |  | 11645 | ND | OS | Farm 1 |
| CC083 |  | 1142 | 828 | OS | Farm 1 |
| CC084 |  | 854 | 828 | OS | Farm 1 |
| CC085 |  | 854 | 828 | AT | Farm 1 |
| CC086 |  | 1122 | 828 | OS | Farm 1 |
| CC087 |  | 854 | 828 | OS | Farm 1 |
| CC088 |  | 854 | 828 | HAT | Farm 1 |
| CC089 |  | 11645 | ND | AT | Farm 1 |
| CC090 |  | 1142 | 828 | OS | Farm 2 |
| CC091 |  | 1142 | 828 | OS | Farm 2 |
| CC092 |  | 2699 | 828 | OS | Farm 2 |
| CC093 |  | 1142 | 828 | OS | Farm 2 |
| CC094 |  | 1142 | 828 | OS | Farm 2 |
| CC095 |  | 887 | 828 | AT | Farm 2 |
| CC096 |  | 887 | 828 | AT | Farm 2 |
| CC097 |  | 1556 | 828 | OS | Farm 5 |
| CC098 |  | 10826 | ND | OS | Farm 5 |
| CC099 |  | 890 | 828 | AT | Farm 5 |
| CC100 |  | 10879 | 828 | OS | Farm 5 |
| CC101 |  | 1068 | 828 | OS | Farm 5 |
| CC102 |  | 1556 | 828 | OS | Farm 5 |
| CC103 | Pregnant sows | 8517 | 828 | OS | Farm 3 |
| CC104 |  | 2699 | 828 | OS | Farm 3 |
| CC105 |  | 887 | 828 | OS | Farm 3 |
| CC106 |  | 827 | 828 | AT | Farm 4 |
| CC107 |  | 827 | 828 | AT | Farm 4 |
| CC108 |  | 854 | 828 | OS | Farm 6 |
| CC109 |  | 854 | 828 | OS | Farm 6 |
| CC110 |  | 8517 | 828 | OS | Farm 6 |
| CC111 |  | 2699 | 828 | AT | Farm 1 |
| CC112 |  | 2699 | 828 | HAT | Farm 1 |
| CC113 |  | 10927 | 828 | AT | Farm 1 |
| CC114 |  | 887 | 828 | AT | Farm 1 |
| CC115 |  | 887 | 828 | OS | Farm 1 |
| CC116 |  | 1058 | 828 | HAT | Farm 2 |
| CC117 |  | 1058 | 828 | OS | Farm 2 |
| CC118 |  | 1096 | 828 | OS | Farm 2 |
| CC119 |  | 8517 | 828 | HAT | Farm 2 |
| CC120 |  | 1068 | 828 | HAT | Farm 5 |
| CC121 |  | 1068 | 828 | HAT | Farm 5 |
| CC122 |  | 10879 | 828 | HAT | Farm 5 |
| CC123 |  | 1556 | 828 | OS | Farm 5 |
| CC124 |  | 854 | 828 | AT | Farm 5 |

^*^ND, not determined.

^**^OS, oxygen-sensitive; AT, aerotolerant; HAT, hyper-aerotolerant.

**Reference**

Bacon, D. J., R. A. Alm, D. H. Burr, L. Hu, D. J. Kopecko, C. P. Ewing and P. Guerry (2000). "Involvement of a plasmid in virulence of Campylobacter jejuni 81-176." Infection and immunity **68**(8): 4384-4390.

Bang, D. D., E. M. Nielsen, F. Scheutz, K. Pedersen, K. Handberg and M. Madsen (2003). "PCR detection of seven virulence and toxin genes of Campylobacter jejuni and Campylobacter coli isolates from Danish pigs and cattle and cytolethal distending toxin production of the isolates." J Appl Microbiol **94**(6): 1003-1014.

Bang, D. D., F. Scheutz, P. Ahrens, K. Pedersen, J. Blom and M. Madsen (2001). "Prevalence of cytolethal distending toxin (cdt) genes and CDT production in Campylobacter spp. isolated from Danish broilers." J Med Microbiol **50**(12): 1087-1094.

Bleumink-Pluym, N. M., L. B. van Alphen, L. I. Bouwman, M. M. Wösten and J. P. van Putten (2013). "Identification of a functional type VI secretion system in Campylobacter jejuni conferring capsule polysaccharide sensitive cytotoxicity." PLoS Pathog **9**(5): e1003393.

Corcionivoschi, N., O. Gundogdu, L. Moran, C. Kelly, P. Scates, L. Stef, A. Cean, B. Wren, N. Dorrell and R. H. Madden (2015). "Virulence characteristics of hcp+ Campylobacter jejuni and Campylobacter coli isolates from retail chicken." Gut pathogens **7**(1): 20.

Datta, S., H. Niwa and K. Itoh (2003). "Prevalence of 11 pathogenic genes of Campylobacter jejuni by PCR in strains isolated from humans, poultry meat and broiler and bovine faeces." J Med Microbiol **52**(Pt 4): 345-348.

Gonzalez, I., K. A. Grant, P. T. Richardson, S. F. Park and M. D. Collins (1997). "Specific identification of the enteropathogens Campylobacter jejuni and Campylobacter coli by using a PCR test based on the ceuE gene encoding a putative virulence determinant." J Clin Microbiol **35**(3): 759-763.

Konkel, M. E., S. A. Gray, B. J. Kim, S. G. Garvis and J. Yoon (1999). "Identification of the enteropathogens Campylobacter jejuni and Campylobacter coli based on the cadF virulence gene and its product." J Clin Microbiol **37**(3): 510-517.

Linton, D., A. J. Lawson, R. J. Owen and J. Stanley (1997). "PCR detection, identification to species level, and fingerprinting of Campylobacter jejuni and Campylobacter coli direct from diarrheic samples." J Clin Microbiol **35**(10): 2568-2572.

Linton, D., R. J. Owen and J. Stanley (1996). "Rapid identification by PCR of the genus Campylobacter and of five Campylobacter species enteropathogenic for man and animals." Res Microbiol **147**(9): 707-718.

Müller, J., F. Schulze, W. Müller and I. Hänel (2006). "PCR detection of virulence-associated genes in Campylobacter jejuni strains with differential ability to invade Caco-2 cells and to colonize the chick gut." Vet Microbiol **113**(1-2): 123-129.

Nachamkin, I., H. Ung and C. M. Patton (1996). "Analysis of HL and O serotypes of Campylobacter strains by the flagellin gene typing system." J Clin Microbiol **34**(2): 277-281.
